# *Pmch*-Deficiency in Rats Is Associated with Normal Adipocyte Differentiation and Lower Sympathetic Adipose Drive

**DOI:** 10.1371/journal.pone.0060214

**Published:** 2013-03-26

**Authors:** Joram D. Mul, Eoghan O’Duibhir, Yogendra B. Shrestha, Arjen Koppen, Peter Vargoviç, Pim W. Toonen, Eleen Zarebidaki, Richard Kvetnansky, Eric Kalkhoven, Edwin Cuppen, Timothy J. Bartness

**Affiliations:** 1 Hubrecht Institute-KNAW and University Medical Center Utrecht, Utrecht, The Netherlands; 2 Department of Biology, Neurobiology and Behavior Program, and Exploring and Testing Strategies for Obesity Reversal Center, Georgia State University, Atlanta, Georgia, United States of America; 3 Department of Metabolic and Endocrine Diseases, University Medical Center Utrecht, Utrecht, The Netherlands; 4 Laboratory for Stress Research, Institute of Experimental Endocrinology, Bratislava, Slovakia; 5 Department of Medical Genetics, University Medical Center Utrecht, Utrecht, The Netherlands; Kent State University, United States of America

## Abstract

The orexigenic neuropeptide melanin-concentrating hormone (MCH), a product of *Pmch*, is an important mediator of energy homeostasis. *Pmch*-deficient rodents are lean and smaller, characterized by lower food intake, body-, and fat mass. *Pmch* is expressed in hypothalamic neurons that ultimately are components in the sympathetic nervous system (SNS) drive to white and interscapular brown adipose tissue (WAT, iBAT, respectively). MCH binds to MCH receptor 1 (MCH1R), which is present on adipocytes. Currently it is unknown if *Pmch*-ablation changes adipocyte differentiation or sympathetic adipose drive. Using *Pmch*-deficient and wild-type rats on a standard low-fat diet, we analyzed dorsal subcutaneous and perirenal WAT mass and adipocyte morphology (size and number) throughout development, and indices of sympathetic activation in WAT and iBAT during adulthood. Moreover, using an *in vitro* approach we investigated the ability of MCH to modulate 3T3-L1 adipocyte differentiation. *Pmch*-deficiency decreased dorsal subcutaneous and perirenal WAT mass by reducing adipocyte size, but not number. In line with this, *in vitro* 3T3-L1 adipocyte differentiation was unaffected by MCH. Finally, adult *Pmch*-deficient rats had lower norepinephrine turnover (an index of sympathetic adipose drive) in WAT and iBAT than wild-type rats. Collectively, our data indicate that MCH/MCH1R-pathway does not modify adipocyte differentiation, whereas *Pmch*-deficiency in laboratory rats lowers adiposity throughout development and sympathetic adipose drive during adulthood.

## Introduction

Adipocytes not only store excess energy (as triglycerides), but also function as endocrine cells that take part in the regulation of energy homeostasis. Obesity, characterized by excess energy stored in white adipose tissue (WAT), reflects the cumulative sum of the excess energy intake over energy expenditure over time [1]. The sympathetic nervous system (SNS) innervates both WAT and interscapular brown adipose tissue (iBAT), and is the primary initiator of lipolysis through its principal catecholaminergic neurotransmitter norepinephrine [2], [3], [4], [5]. Thus, in general, chronic sympathetic activity increases the fuel availability through increased lipid mobilization in WAT. Several central nervous system (CNS) circuits modulate SNS outflow to WAT, including leptin [6], insulin [7], [8], ghrelin [9], neuropeptide Y [10], and melanocortins [11], all of which affect adipose metabolism.

The hypothalamic orexigenic neuropeptide melanin-concentrating hormone (MCH), derived from *Pmch*, is an important effector of energy intake and expenditure [12], [13], [14]. *Pmch*-deficient rats are lean, characterized by lower food intake, body- and fat mass, and decreased energy expenditure [15]. MCH binds to MCH receptor 1 (MCH1R) [16], [17], [18]. Rodents only express MCH1R, whereas humans also express a second MCH receptor, MCH2R [19].

Retrograde viral transneuronal tract tracing studies have identified *Pmch* neurons in the lateral hypothalamus amongst other neurons as part of the central SNS outflow to WAT and iBAT [20], [21], [22]. Furthermore, MCH1Rs are expressed in isolated rat adipocytes and murine 3T3-L1 (pre)-adipocytes [23], [24]. In 3T3-L1 (pre)-adipocytes, MCH facilitates migration [25], activates signaling pathways [24], and regulates leptin synthesis [23]. On the contrary, MCH has no direct effect on adipocyte lipolysis [26]. Recently, an elegant study demonstrated that central activation of MCH1R in wild-type rats increases fat deposition in WAT via suppression of sympathetic drive [27].

Currently it is unknown whether chronic loss of MCH-signaling affects adipocyte differentiation or sympathetic adipose drive. Using *Pmch*-deficient [15] and wild-type control rats, we analyzed dorsal subcutaneous and perirenal WAT mass and adipocyte morphology throughout development. In addition, using an *in vitro* approach we investigated the ability of MCH to modulate 3T3-L1 adipocyte differentiation. Finally, norepinephrine turnover was measured as an index of sympathetic adipose drive during adulthood. Our data reveal that the MCH/MCH1R-pathway has no or a very minor role in white adipocyte differentiation Finally, our findings reveal that *Pmch*-deficiency in the rat lowers sympathetic adipose drive. The latter adaptation potentially favors adipose lipid deposition during the negative energy balance resulting from *Pmch*-deficiency.

## Materials and Methods

### Ethics Information

The Animal Care Committee of the Royal Dutch Academy of Science approved all experiments according to the Dutch legal ethical guidelines.

### Rats, Housing, and Diet

Experimental wild-type (WT) and *Pmch*-deficient (HOM) littermate rats were generated using a heterozygous breeding strategy [15]. Two rats were housed together, unless noted otherwise, under controlled experimental conditions (12 h light/dark cycle, light period 0600–1800, 21±1°C, ∼60% relative humidity). A standard chow diet (RM3; 62% kcal from carbohydrate, 27% kcal from protein, 11% kcal from fat, 3.33 kcal/g AFE, SDS, Witham, United Kingdom) and water were provided *ad libitum* unless noted otherwise. All rats had access to home-cage enrichment (red rat retreat [Plexx, Elst, the Netherlands] and aspen gnaw brick [Technilab-BMI, Someren, the Netherlands]), unless noted otherwise. Genotyping was done as previously described [15], and genotypes were reconfirmed when experimental procedures were completed. Only male rats were used in this study.

### WAT Mass and Morphology

After measuring body mass, rats were sacrificed at PND 40, 60, or 120 by asphyxiation followed by decapitation. The left and right dorsal subcutaneous WAT (dWAT) and perirenal (pWAT) fat pads, the left lateral liver lobe (as a indication of liver mass), and the adrenal glands were isolated and weighed, and adipose tissue volume was measured. WAT samples were collected in 4% formaldehyde, rotated overnight at room temperature, rinsed with 70% ethanol for 2 h, 96% ethanol for 2 h, twice with 100% ethanol for 1.5 h, 2 h in xylene, and embedded in paraffin overnight. Samples were then cut in 14 *µ*m sections and stained with eosin. Ten sections/animal were cut at positions distributed equally throughout the WAT pad to minimize for local effects, and one picture was obtained per section. Images were captured with an Axioplan 2 microscope (Zeiss). Subsequently, average adipocyte cell diameter was measured using NIH ImageJ software. Using the average adipocyte cell diameter, adipose tissue volume, and correcting for the percentage of non-adipocyte tissue, an estimated number of total adipocyte cells was calculated. In a separate cohort of rats, after measuring body mass, rats were sacrificed at PND 140 by asphyxiation and decapitation, and iBAT was isolated and weighed.

### Hormone Measurements

WT and HOM rats were sacrificed at PND 40, 60, or 120 by asphyxiation and decapitation, and whole blood was collected. Blood samples were allowed to clot at room temperature for 30 min and centrifuged at 2150 rcf for 15 min at 4°C. Serum samples were then aliquoted and stored at −80°C until analysis. Serum leptin levels were measured in duplicate using a leptin ELISA (EZRL-83K; Linco Research, St. Charles, Missouri, USA), as previously described [15].

### *In vitro* Differentiation of 3T3-L1 Cells and FABP4 Western Blot

Differentiation assays and Western blotting on murine 3T3-L1 cells were performed as described earlier [28], [29]. Antibodies used for Western blot analysis were: anti-FABP4 (sc18661; Santa Cruz Biotechnology, Santa Cruz, CA, USA) and anti-Tubulin (ab6046; Abcam, Cambridge, MA, USA).

### NETO and Tissue Preparation

NE turnover (NETO; an index of sympathetic activation) was measured using the α-methyl-p-tyrosine (AMPT) method [30], [31]. AMPT methyl ester hydrochloride (Sigma Aldrich, Zwijndrecht, The Netherlands) was freshly prepared by first adding an aliquot of glacial acetic acid (1 *µ*l/mg AMPT) and then diluting to the final concentration with 0.15 M NaCl. The pH was set to 7.0 using 5 M NaOH. At the beginning of the experiment, rats within the same genotype were paired based on body weight. At PND 153, half of the rats were untreated and sacrificed at t = 0 h to obtain baseline tissue NE content, whereas the paired other half was injected intraperitoneal (IP) with AMPT (250 mg AMPT per kilogram; 25 mg/ml) between 0900 and 1200. AMPT-treated rats received a second IP AMPT dose (125 mg/kg body mass; 12.5 mg/ml) 2 h after the initial AMPT injection to assure the maintenance of tyrosine hydroxylase inhibition. AMPT-treated rats were sacrificed 4 h after the initial AMPT injection by decapitation without anesthesia, after which dWAT, pWAT, epididymal WAT (eWAT), and iBAT were rapidly dissected, weighed, frozen in liquid nitrogen, and stored at −80°C until assayed for NE content by high pressure liquid chromatography to determine NE content and NETO was subsequently calculated as previously [30]. Before the NETO experiment, rats were handled and sham-injected daily during 7 d to minimize handling/stress-induced increases in SNS activity during the experiment.

### *Ucp1* and *Adrb3* mRNA Expression

iBAT mRNA expression was analyzed as previously described [15], using the following primers: uncoupling protein-1 (*Ucp1*), F: TCAGC TTTGC TTCCC TCAGG ATTG, R: AGCCG AGATC TTGCT TCCCA AAGA; β3-adrenoceptor (*Adrb3*), F: AGTCC ACCGC TCAAC AGGTT TGAT, R: AGCTT CCTTG CTGGA TCTTC ACG.

### Data Analysis

Data are shown as mean ± S.E.M. All data were analyzed using a commercially available statistical program (SPSS for Macintosh, version 16.0) and were controlled for normality and homogeneity. All data were analyzed using a two-tailed Student’s *t*-test. The null hypothesis was rejected at the 0.05 level.

## Results

### Chronic *Pmch*-deficiency Lowers Adipose Mass During Rat Development

**Figure 1:**
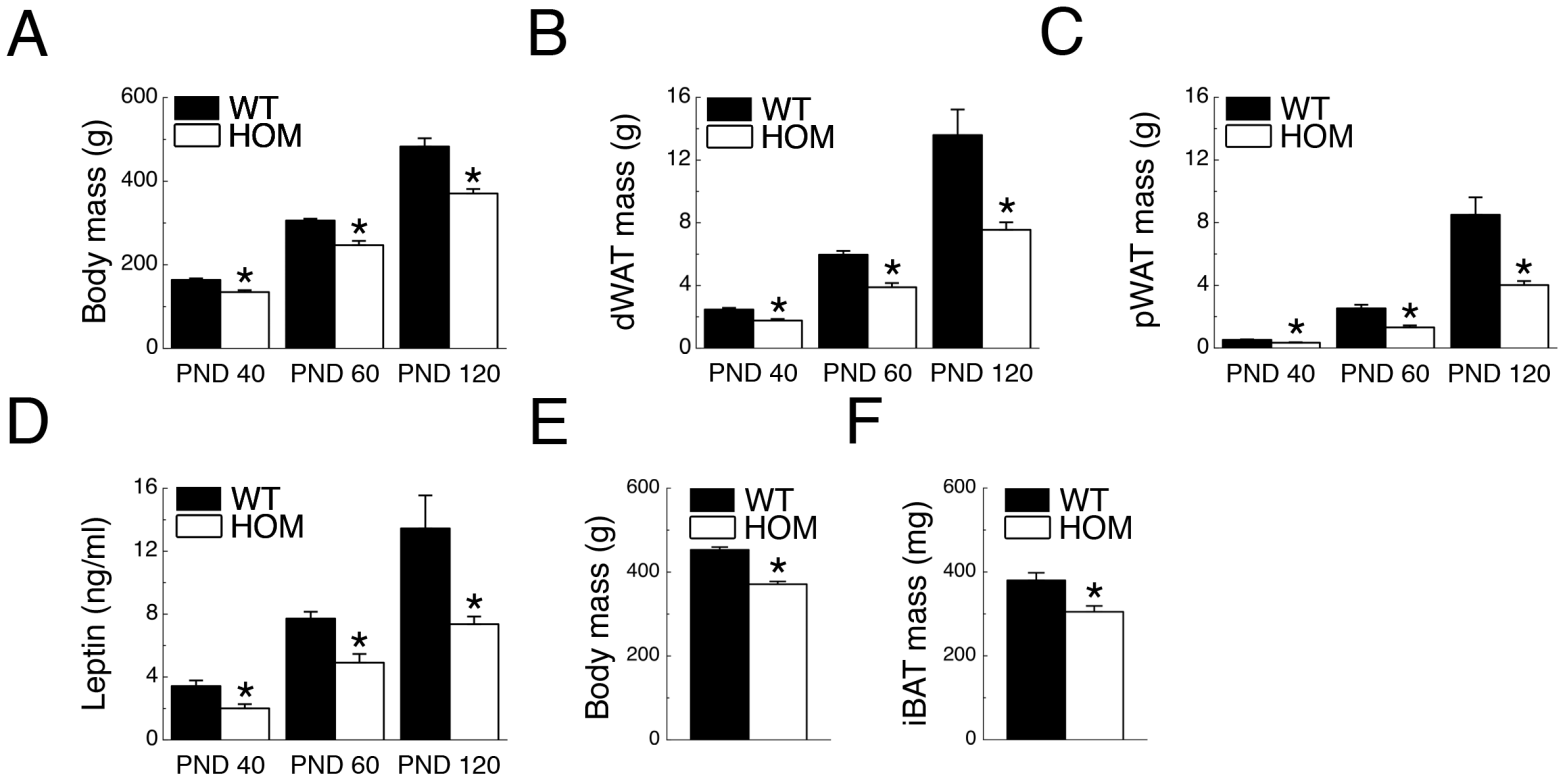
*Pmch*-deficiency lowers body- and fat mass during development. (**A**) Body mass (BM), (**B**) dWAT mass, (**C**) pWAT mass, and (**D**) serum leptin concentrations in WT and HOM rats (n = 8–15/group) at postnatal (PND) days 40, 60, and 120, and (**E**) body mass and (**F**) iBAT mass in WT and HOM rats (n = 15–19/group) at PND 147. *P<0.05 vs WT.

Body-, dWAT-, and pWAT mass was analyzed at PND 40, 60, and 120. At all three time-points, HOM rats had lower body mass than WT rats (*P*s <0.05; Fig. 1A). Mirroring body mass, HOM rats had lower dWAT and pWAT mass than WT rats at all three time-points (*P*s <0.05; Figs. 1B and 1C, respectively). HOM rats had lower serum leptin concentrations than WT rats at PND 40, 60, and 120, mirroring lower adiposity levels (*P*s <0.05; Fig. 1D). In a separate cohort we analyzed iBAT mass at PND 140. HOM rats had lower body- and iBAT mass than WT rats (*Ps* <0.05; Figs. 1E and 1F).

### Chronic *Pmch*-deficiency Decreases Adipocyte Cell Size *in vivo*

**Figure 2:**
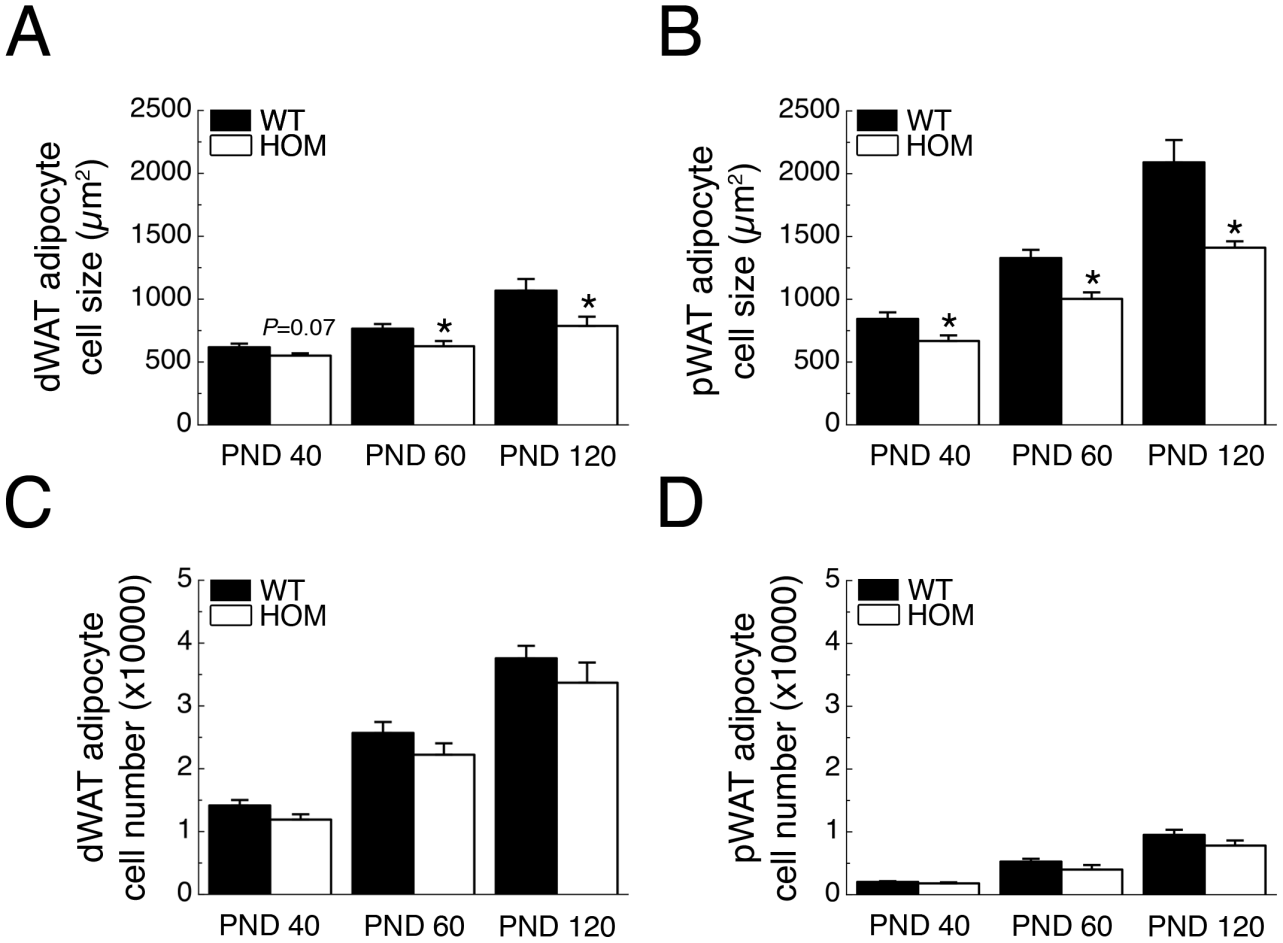
*Pmch*-deficiency decreases white adipocyte size. (**A**) dWAT- (**B**) and pWAT adipocyte cell size, (**C**) dWAT- and (**D**) pWAT adipocyte cell number of WT and HOM rats (n = 8–15/group) at PND 40, 60, and 120. *P<0.05 vs WT.

HOM rats had lower WAT mass than WT rats, which can result from changes in adipocyte size, cell number or both. Therefore, we analyzed dWAT and pWAT adipocyte cell size and number in WT and HOM rats at PND 40, 60, and 120. HOM rats had smaller dWAT and pWAT adipocyte cell size than WT rats at PND 40, 60 and 120 (*P*s <0.05; except for dWAT PND40, *P = *0.07; Figs. 2A and 2B, respectively). dWAT and pWAT adipocyte cell number, however, did not differ significantly between genotypes at PND 40, 60 and 120 (*P*s >0.05; Figs. 2C and 2D, respectively).

### MCH does not Affect 3T3-L1 Adipocyte Differentiation *in vitro*

**Figure 3:**
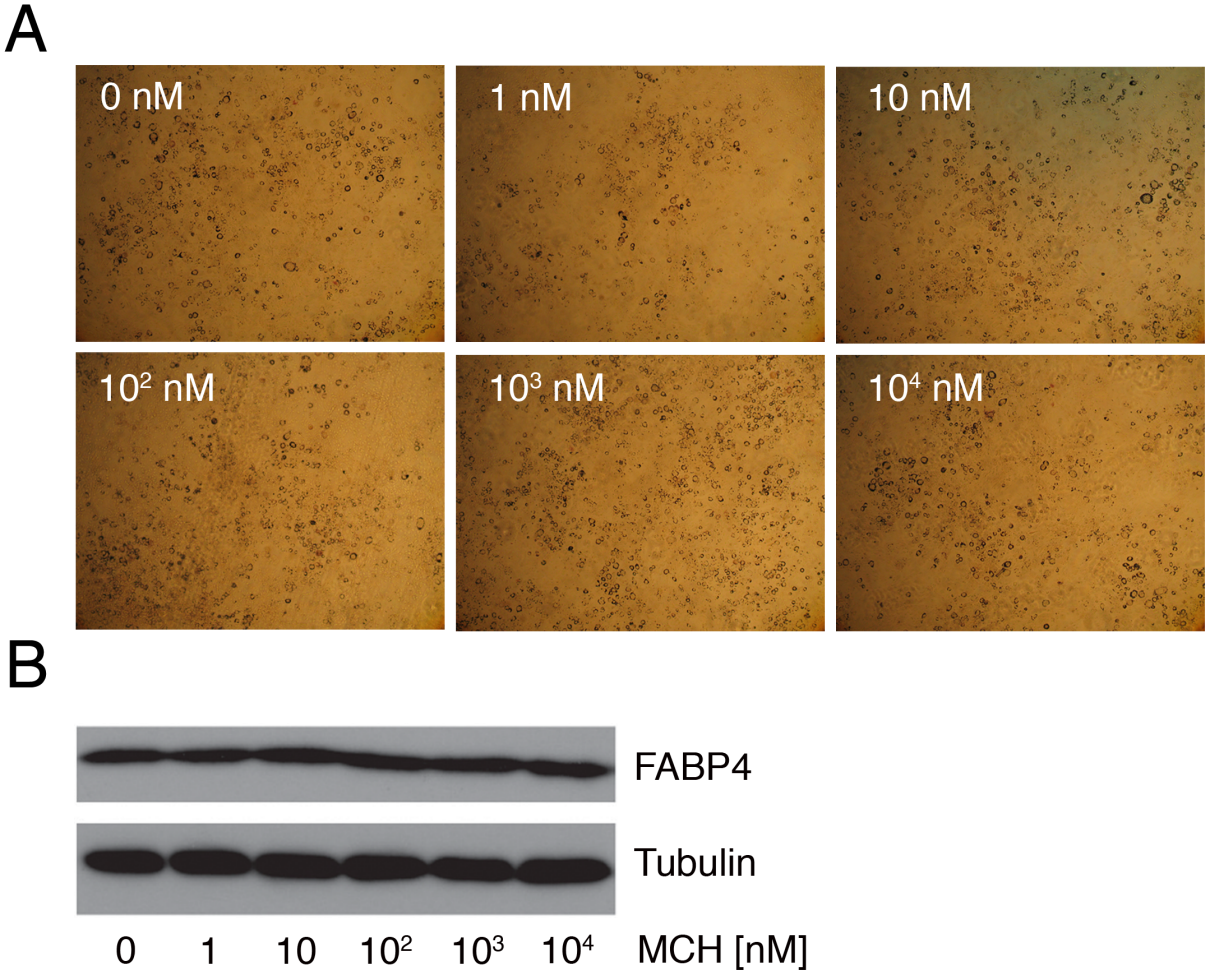
MCH does not modify *in vitro* 3T3-L1 cell differentiation. (**A**) 3T3-L1 cell differentiation after 7-day administration of 1 nM, 10 nM, 100 nM, 1 *µ*M, or 10 *µ*M MCH as visualized by Oil-red-O staining (x20 magnification). (**B**) Western blot analysis for FABP4 (also known as aP2). Tubulin was used to control for input. Assays were performed in triplicate.

MCH1Rs are expressed in isolated rat adipocytes and murine 3T3-L1 (pre)-adipocytes [23], [24]. Therefore, we investigated if MCH can modulate 3T3-L1 adipocyte differentiation *in vitro*. Treatment of differentiating 3T3-L1 cells with MCH (1 nM, 10 nM, 100 nM, 1 *µ*M, or 10 *µ*M) had no visible effects on adipocyte differentiation as visualized by Oil-red-O staining (Fig. 3A). Furthermore, protein analysis for FABP4, an indicator of adipocyte differentiation [32], confirmed no effect of MCH on 3T3-L1 adipocyte differentiation *in vitro* (Fig. 3B).

### Chronic *Pmch*-deficiency Decreases Sympathetic Drive to Adipose Tissue

**Figure 4:**
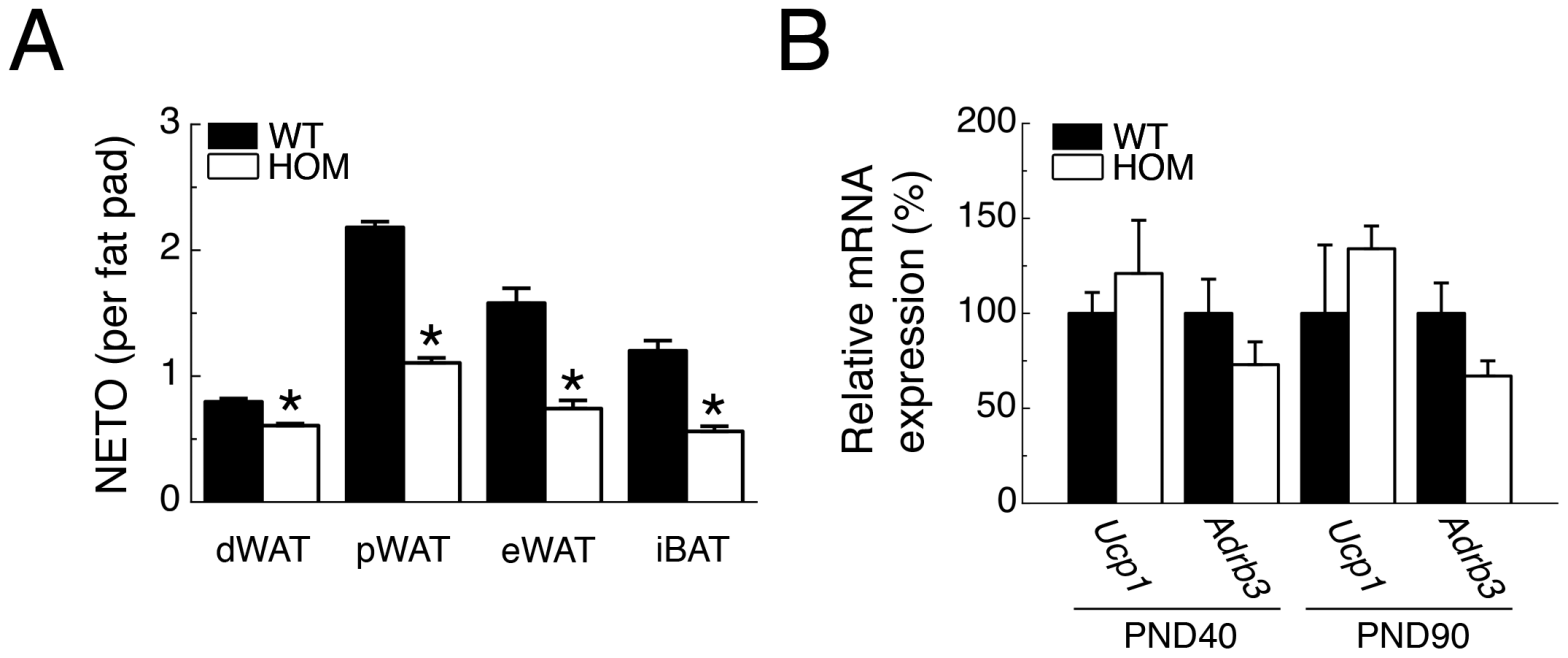
*Pmch*-deficiency lowers sympathetic adipose drive. (**A**) NETO in dWAT, pWAT, eWAT, and iBAT of WT and HOM rats at PND 153 (n = 8–11/group). (**B**) Relative mRNA expression of Uncoupling Protein 1 (*Ucp1*) and β3-adrenoceptor (*Adrb3*) in iBAT of WT and HOM rats at PND 40 (left) and 90 (right) (n = 5–6/group). *P<0.05 vs WT.

Retrograde viral transneuronal tract tracing studies have identified *Pmch* neurons in the lateral hypothalamus amongst other neurons as part of the central SNS outflow to WAT or iBAT [20], [21], [22]. Therefore we investigated if *Pmch*-deficiency changes the sympathetic drive to adipose tissue by analyzing NETO, an index of sympathetic activation. NETO is presented on a whole-organ basis to reflect the overall sympathetic drive and physiological impact for each tissue. HOM rats had lower NETO than WT rats in dWAT, pWAT, eWAT, and IBAT (Fig. 4A). We also analyzed mRNA expression levels of uncoupling protein-1 (*Ucp1*) and the β3-adrenoceptor (*Adrb3*) in iBAT, which did not differ significantly between genotypes at PND40 or 90 (Fig. 4B).

## Discussion

The present data demonstrate that chronic loss of MCH-signaling in the rat lowers adiposity throughout development and sympathetic adipose drive during adulthood, whereas adipocyte differentiation was unaffected during development.

Our observations that adult HOM rats have lower body mass, adiposity, and serum leptin levels than WT rats are in agreement with earlier observations [15]. Furthermore, we now demonstrate that these changes are already present in HOM rats at PND 40 and 60. Because HOM rats have decreased WAT mass and MCH1Rs are expressed on adipocytes, we investigated whether *Pmch*-deficiency had an effect on adipocyte number and size. *In vivo*, we observed robust differences in WAT adipocyte cell size, but no significant differences in adipocyte cell number, and *in vitro* we observed no effect of MCH administration on 3T3-L1 differentiation. In sum, our data suggest no major role for MCH/MCH1R-mediated signaling in adipocyte differentiation. In humans, however, this does not exclude a functional role for MCH/MCH2R-mediated signaling, as MCH2R is present in adipose tissue at significant levels and modulates adipocyte differentiation during an *in vitro* cell-based approach [33], [34].

A key observation of the present study is that adult HOM rats have lower sympathetic adipose drive, which corresponds with earlier observations that HOM rats have lower energy expenditure [15]. A decrease in energy expenditure could result from the hypophagic character of HOM rats [15], [35], as acute MCH1R agonism only affects energy expenditure in a feeding-dependent manner [36]. The SNS controls adipocyte lipolysis and several other metabolic responses through norepinephrine-mediated activation of the β3-adrenoceptor (*Adrb3*) [37]. Here we observed that iBAT *Ucp1* expression, which is downstream of β3-adrenoceptor activation, was not significantly changed between genotypes at PND40 and 90. At these time points iBAT *Adrb3* expression was slightly lower in HOM rats compared to WT rats, although not significantly, which is in line with the lower sympathetic drive to iBAT [38].

Recently it was demonstrated that 7-day intracerebroventricular infusion of MCH in wild-type rats increases fat deposition in WAT via suppression of sympathetic drive [27]. Surprisingly, despite different experimental approaches (chronic loss of *Pmch* versus pharmacological central administration of MCH), this finding supports the lower sympathetic adipose drive observed in the present study. Because MCH stimulates caloric intake [13], it is not unexpected that central MCH administration also stimulates lipid storage through direct modulation of adipocyte metabolism [27], overall promoting a positive energy balance. *Pmch*-deficiency, however, results in a negative energy balance and a similar reduction of sympathetic adipose drive. We suggest that the latter adaptation potentially favors adipose lipid deposition during this negative energy balance. Furthermore, chronic absence of MCH-signaling in our rat model has significant effects on physiology, including lower leptin levels in the circulation (this study and [15]) and lower hypothalamic *Pro-opiomelanocortin* (*Pomc*) mRNA expression during adulthood [15]. Central leptin signaling in the mediobasal hypothalamus can inhibit WAT lipogenesis through a sympathetic route [6], and activation of central melanocortin receptors increases iBAT NETO [30]. Thus, reduced central leptin- and melanocortin signaling could contribute to the lower adipose SNS activity in HOM rats, aiding in the promotion of a positive energy balance.

The orexigenic hypothalamic neuropeptide MCH/MCH1R-system has been the subject of many studies (for review see: [13], [14]), as functional inhibition of MCH, MCH1R, or a combination of both might result in an anti-obesity treatment [39]. Here we show in the rat that *Pmch*-deficiency lowers sympathetic adipose drive during adulthood. Understanding the mechanisms how *Pmch*-deficiency changes the autonomic balance might further help the development of a potential anti-obesity treatment based on the MCH/MCH1R system.
